# A Computational Approach for Modeling the Allele Frequency Spectrum of Populations with Arbitrarily Varying Size

**DOI:** 10.1016/j.gpb.2019.06.002

**Published:** 2020-03-13

**Authors:** Hua Chen

**Affiliations:** 1 CAS Key Laboratory of Genomic and Precision Medicine, Beijing Institute of Genomics, Chinese Academy of Sciences, Beijing 100101, China; 2 CAS Center for Excellence in Animal Evolution and Genetics, Chinese Academy of Sciences, Kunming 650223, China; 3 School of Future Technology, University of Chinese Academy of Sciences, Beijing 100049, China

**Keywords:** Allele frequency spectrum, Complex demography, Population history, Population genetic inference, Coalescent

## Abstract

The **allele frequency spectrum** (AFS), or site frequency spectrum, is commonly used to summarize the genomic polymorphism pattern of a sample, which is informative for inferring **population history** and detecting natural selection. In 2013, Chen and Chen developed a method for analytically deriving the AFS for populations with temporally varying size through the coalescence time-scaling function. However, their approach is only applicable to population history scenarios in which the analytical form of the time-scaling function is tractable. In this paper, we propose a computational approach to extend the method to populations with arbitrary complex varying size by numerically approximating the time-scaling function. We demonstrate the performance of the approach by constructing the AFS for two population history scenarios: the logistic growth model and the Gompertz growth model, for which the AFS are unavailable with existing approaches. Software for implementing the algorithm can be downloaded at http://chenlab.big.ac.cn/software/.

## Introduction

The allele frequency spectrum (AFS, aka, the site frequency spectrum) is a series of fundamental statistics for summarizing genomic polymorphism. It is defined as the sampling distribution of allele frequencies of genetic polymorphism in a finite sample. In practice, AFS can be the number or proportion of SNPs constructed by binning them according to the counts of derived alleles. For a sample of n sequences with m identified segregating sites (polymorphic sites), AFS is written as $\left\{\left(S_{i}\right),\text{\textbackslash{};}1⩽i<n\right\}$, with $\sum_{i=1}^{n-1}S_{i}=m$, where $S_{i}$ denotes the number of segregating sites in the sample that have i copies of derived alleles among the n haplotypes. AFS has been a main focus in theoretical and methodological studies in the past decades, since it is informative for the inference of ancient demography of populations [1]. The theoretical expectation of AFS under a given population history and parameter setting can be developed using both coalescent theory and diffusion [2], [3], [4]. Methods for ancestral inference based on AFS are then developed in a Poisson random field framework by assuming that each entry of the AFS follows a Poisson distribution with the mean equal to the theoretical expectation of the AFS given population genetic parameters [2], [3], [4], [5], [6], [7], [8], [9], [10], [11], [12], [13], [14], [15], [16], [17]. These methods are gaining popularity with the abundance of genomic sequencing data.

Coalescent theory has been applied to developing AFS in a single population with time-varying population sizes, including the exponential-growth model [7], [18] and the n-epoch model, which models the population size changes using several consecutive periods (epochs) with different constant sizes [8]. Compared with the AFS developed with diffusion, the coalescent-based AFS has the advantage of being in analytical form, and the estimation is fast and accurate for small samples. In contrast, the diffusion approximation has to rely on numerical methods, such as finite difference approaches, to approximate the solutions [9], [19]. The coalescent-based AFS is thus very useful for the inference of past demographic history and has been extensively applied to data analysis [20], [21], [22], [23], [24].

One limitation of the coalescent-based AFS methods is that AFS can only be analytically derived for some simple population growth models, such as the n-epoch model and the exponential-growth model or their combinations thereof [23], [7], [8], and generalizations to other complex population histories are often impracticable [7], [13], [25]. A second limitation is that for large samples (*e.g.*, haplotype number n>50), it is hard to accurately calculate the expected AFS from the formulae. The expected coalescence times $ET_{i},\;1⩽i<n$ are essential for deriving the coalescent-based AFS, which contain coefficients in the alternating sum of the hypergeometric series and are explosively large, causing overflow for large sample sizes [26]. When the sample size is large, AFS and its derived statistics are informative for inferring recent population history. And thus, calculating AFS for large samples becomes common in population genetic inference from genomic data [23], [27], [28]. A high-precision arithmetic library is usually adopted to obtain accurate numerical values when analyzing larger samples, which requires tedious programming and intensive computation [8]. Some alternative solutions were proposed, specifically for AFS of a single population, *e.g.*, Polanski and Kimmel [7] replaced it with hypergeometric summation to avoid estimating coefficients with large values. Their approach can efficiently solve the numerical issue, but it is difficult to generalize to other scenarios with complex population histories for which the integral function in the hypergeometric summation is difficult to compute. Most studies have adopted coalescent simulations to generate a large number of samples to approximate AFS under specific demographic histories and applied them to analyze genomic polymorphism. However, this approach is computationally very intensive [14], [23], [27], [29], [30], [31].

To address the numerical issue in large samples, Chen and Chen used the large-sample asymptotic distributions of coalescence times [32]. Griffiths proved that the coalescence times and ancestral lineage numbers asymptotically follow a normal distribution in a constant population [33]. Chen and Chen extended their forms to populations with time-varying sizes by using a time-scaling function scheme (see the “Coalescence times” subsection below; [34], [35], [36]) and then used the first-order Taylor expansion approximation to achieve the coalescence times (and further AFS). They illustrated the usage of this approach by deriving a simple-form formula for AFS in populations under exponential growth, which shows high accuracy compared with simulated results. Note that the first-order Taylor expansion approximation and time-scaling function approach of Chen and Chen work for both large and small size samples [32]. Technically, their approach allows to derive AFS in any populations with arbitrary complex demography. However, as illustrated in the “Method” section, for some complex demographies, it is difficult to derive the analytical form of the time-scaling function and/or its inverse function, which are essential in deriving the coalescent-based AFS. In this paper, we propose a computational approach to efficiently approximate the analytical formula of the time-scaling function with a finite sum approximation, and find the set of coalescence times $ET_{i},\;1⩽i<n$, with the computing time being nearly constant as the sample size increases. It is applicable to any arbitrary complex history for which the time-scaling function is not tractable. This greatly extends the application of AFS-based methods in population genetic inference and other studies, *e.g.*, cancer evolution. We demonstrate the performance of the approach by obtaining AFS for two population history scenarios that were difficult to derive using the existing approaches: the logistic growth model and the Gompertz model.

In the following sections we first review the coalescent theory framework for obtaining AFS of a single population. We then summarize the first-order Taylor expansion approximation method for populations with time-varying size proposed by Chen and Chen [32]. We illustrate the idea of the computational approach to construct AFS for arbitrary demography, and we further derive AFS for populations with two demographic histories to demonstrate its performance.

## Method

### Modeling framework

For a sample of n lineages (haplotypes), the coalescence time $T_{k}$ is defined as the time when k+1 lineages merge into k lineages, and time is measured backward (from the present to the past). The intercoalescence time $W_{k}=T_{k-1}-T_{k}$ is the time during which there are k lineages. Following Fu [2], we say that any of the k branches spanning the intercoalescence time $W_{k}$ has the branch of size k. We assume an infinitely-many-sites model for mutations, and further the mutations occurring on any branch along the gene genealogy follow a Poisson process. The number of mutations occurring at any branch of size k then follows a Poisson distribution with the mean of $\mu kE\left(W_{k}\right)$, where μ is the point mutation rate. During the bifurcation process in which k lineages increase to n lineages at present, any of these mutations increases the allele count from a single copy to j among the n lineages with the probability [3], [37]:

(1) $$p_{n,k}\left(j\right)=\frac{\left(\begin{array}{c} n-j-1\\ k-2 \end{array}\right)}{\left(\begin{array}{c} n-1\\ k-1 \end{array}\right)}.$$

Summing over mutations that occur on branches with different sizes, we can obtain the entries for AFS:

(2) $$ES_{j}\left(n\right)=\sum_{k=2}^{n}\frac{\left(\begin{array}{c} n-j-1\\ k-2 \end{array}\right)}{\left(\begin{array}{c} n-1\\ k-1 \end{array}\right)}\mu \times kE\left(W_{k}\right)=\frac{\left(n-j-1\right)!\left(j-1\right)!}{\left(n-1\right)!}\mu \sum_{k=2}^{n}k\left(k-1\right)\times \left(\begin{array}{c} n-k\\ j-1 \end{array}\right)E\left(W_{k}\right),\;0<j<n$$

Note that $EW_{j}$ is fundamental in the framework above for constructing AFS. If analytical forms for $EW_{j}=ET_{j-1}-ET_{j}$ can be obtained for a population with complex demography, AFS can be obtained through Equation (2).

### Coalescence times

In a constant-size population, the distribution of coalescence times follows that of the standard Kingman’s n-coalescent, which are exponential variables with the mean

(3) $$\mu_{m}=2\left(\frac{1}{m}-\frac{1}{n}\right),\;1⩽m<n$$

where $\mu_{m}$ is the coalescence time in units of haploid population size N. In addition, the intercoalescence times are mutually independent.

For a population with time-varying size, we denote its population history as $N\left(t\right),t\in \left[0,\infty \right)$. It is not trivial to derive the distribution or the expectation of coalescence times for a population with time-varying sizes. The joint distribution of coalescence times $\left(T_{m},\ldots ,T_{n-1}\right)$ for populations with time-varying size is calcluated as described previously [3] and shown as follows.

(4) $$f_{T_{m},...,T_{n-1}}\left(t_{m},...,t_{n-1}\right)=\prod_{k=m}^{n-1}\frac{\left(\begin{array}{c} k+1\\ 2 \end{array}\right)}{N_{0}\lambda \left(t_{k}\right)}\exp\left(-\frac{\left(\begin{array}{c} k+1\\ 2 \end{array}\right)}{N_{0}}\int_{t_{k-1}}^{t_{k}}\frac{1}{\lambda \left(u\right)}du\right),$$

where $\lambda \left(t\right)=N\left(t\right)/N_{0}$ is the relative size function. Polanski et al. derived the marginal probability density function of coalescence times $f_{T_{m}}$ by expanding an integral transform of the marginal probability density function (PDF) into partial fractions [26]. Another way to derive $f_{T_{m}}$ is based on the definition of a pure-death process, in the form of a function of the ancestral lineage number, $P\left(A_{n}\left(t\right)=m\right)$ [13], [38]. With the marginal distribution of coalescence times derived, Polanski and Kimmel obtained AFS for a population under exponential growth, which is in complex form, and requires calculating the hypergeometric series and exponential integral [7].

Chen and Chen [32] used the time rescaling approach in the variable-population-size coalescent model [34], [35], [36]. The coalescence time is rescaled at the rate $1/N\left(t\right)$, denoted as $\tau_{m}$:

(5) $$\tau_{m}=g\left(T_{m}\right)=\int_{0}^{T_{m}}\frac{1}{N\left(u\right)}du,$$

where $\tau_{m}$ follows the coalescence time distribution in the standard Kingman’s n- coalescent [39]. Chen and Chen [32] then used a Taylor expansion of $T_{m}=g^{-1}\left(\tau_{m}\right)$ around $\mu_{m}$ to achieve the approximation:

(6) $$T_{m}=g^{-1}\left(\mu_{m}\right)+{\left(g^{-1}\right)}^{\prime }\left(\mu_{m}\right)\left(g\left(T_{m}\right)-\mu_{m}\right)+\frac{{\left(g^{-1}\right)}^{\prime \prime }\left(\mu_{m}\right)}{2}{\left(g\left(T_{m}\right)-\mu_{m}\right)}^{2}+O\left({\left(g\left(T_{m}\right)-\mu_{m}\right)}^{3}\right)$$

Thus we have the mean and variance of $T_{m}$,

(7) $$E\left(T_{m}\right)\approx g^{-1}\left(\mu_{m}\right),$$

and

(8) $$Var\left(T_{m}\right)=\frac{\sigma_{m}^{2}}{{\left(g^{\prime }\left(g^{-1}\left(\mu_{m}\right)\right)\right)}^{2}}$$

In general, for any population history $N\left(t\right),0⩽t<\infty$, time-scaling function $g\left(t\right)$ can be obtained as in Equation (5), and further $ET_{m}=g^{-1}\left(\mu_{m}\right)$ can be obtained as shown above. Chen and Chen demonstrate the application of this approach using an exponentially growing population as an example [32]. ET for the exponential growth model is in a very simple analytical form:

(9) $$ET_{m}=\frac{1}{\gamma }\ln\left(2N_{0}\gamma \left(1/m-1/n\right)+1\right)$$

with γ is the population growth rate, and the obtained AFS is highly accurate (Figure 6 of [32]).

Since it is not trivial to derive the coalescence times for populations with time-varying size in existing studies, and simulations are usually required as a replacement for most studies [14], [23], [27], [30], [31], Chen and Chen’s [32] approach provides simple and efficient solution to obtaining $ET_{m}$. However, for some complex demographies, the analytical form of the time-scaling function $g\left(t\right)$ and its inverse function, which are essential for deriving $ET_{m}$, are not tractable. This prohibits the general usage of their approach for arbitrary population histories.

### Coalescence times under complex demographic history

In this section, we illustrate how to extend Chen and Chen’s [32] method to be applicable to arbitrary population histories using a computational approach. As we can see from the section above, $g\left(t\right)$ and $g^{-1}\left(t\right)$ are the two essential components for deriving coalescence times for a given population history $N\left(t\right)$ [see Equation (7)]. Note that to obtain $ET_{m}$, the analytical form is not required for calculating an arbitrary point t. In contrast, we only need to find a finite number of $T_{m}$ values that correspond to $\mu_{m},\text{\textbackslash{};\textbackslash{};}1⩽m<n$ and satisfy

(10) $$\mu_{m}=g\left(T_{m}\right).$$

The following two numerical schemes are thus proposed for calculating $ET_{m}$, applicable to different situations. The first approach is generally applicable to all cases, including those for which $g\left(t\right)$ cannot be obtained; the second approach is specifically for the case in which an analytical form of $g\left(t\right)$ is available but $g^{-1}\left(t\right)$ is not tractable.

### Approach 1 (finite-sum approximation)

For a sample of size n under the population history $N\left(t\right),t\in \left[0,\infty \right)$, the integral of the time scaling function equation can be simply approximated using the discrete finite summation:

$$\mu_{m}=g\left(T_{m}\right)=\int_{0}^{T_{m}}1/N,\left(u\right)du,\;1⩽m<n$$

(11) $$\approx \sum_{u=0}^{T_{m}}\cdot \frac{1}{N\left(u\right)}$$

Then, for each $\mu_{m}$, the corresponding expected coalescence times $ET_{m}$ can be obtained during the following sequential summation procedures:

Step 1 Given a series of expected coalescence times under the standard n-Kingman’s coalescent $\mu_{m}=2\left(\frac{1}{m}-\frac{1}{n}\right),\;1⩽m<n$, initialize the procedure from generation 0 (the current generation) with $G=\frac{1}{N\left(0\right)}$.
Step 2 Keep increasing the discrete generation time t, and calculate $G=G+\frac{1}{N\left(t\right)}$ until the value t satisfies $\mu_{n-1}\approx \sum_{u=1}^{t}\frac{1}{N\left(u\right)}$; set $T_{n-1}=t$.
Step 3 Repeat Step 2, and keep increasing t to obtain the rest of the values for $ET_{i},\;n-2⩾i⩾1$.
Step 4 Terminate the process when $ET_{1}$ is obtained.

After $\left\{ET_{m},1⩽m<n\right\}$ is available, AFS can be constructed through Equation (2). The detailed pseudocode for implementing the algorithm is listed in Table 1.

**Table 1:** **Procedures for calculating coalescence times using the finite-sum approximation**

**Algorithm: calculating coalescence times**
**Input:** population history $N\left(t\right),\;0⩽t<\infty$, sample size n.
**Initialize:**$\mu_{i}=2\left(\frac{1}{i}-\frac{1}{n}\right),\;i=1,\;2,\;\text{\textbackslash{};}...,\;n-1$; t=0; $G=\frac{1}{N\left(0\right)}$.
**For***i* = *n* − 1:1
$\mu =\mu_{i};$
**While**G<μ
t=t+1;
$\text{\textbackslash{};}G=G+\frac{1}{N\left(t\right)}$;
**End**
**If**$G-\mu <\frac{1}{N\left(t\right)}$
$ET_{i}=t$;
**Else**
$ET_{i}=t-1$;
**End**
**End**
**Output:** expected coalescence times $ET_{i},\;i=1,\;2,\;...,\;n-1.$

### Approach 2

For some population histories, analytical form of the time scaling function $g\left(t\right)$ can be achieved, but the inverse function $g^{-1}\left(t\right)$ is not tractable. An alternative approach can be applied to obtain $ET_{m}$ for such cases through the following procedures. For each $T_{m},1⩽m<n$, we have the non-linear equation,

(12) $$g\left(T_{m}\right)-\mu_{m}=0,1⩽m<n.$$

The non-linear equations above can be solved using numerical algorithms to obtain $T_{m},$ such as Newton-Raphson [40]. In this paper, we adopt two numerical methods implemented in MATLAB. The first one is the fzero function, which implements Dekker’s algorithm as a combination of bisection, secant, and inverse quadratic interpolation methods [41]. The second is the fminsearch function, which uses the simplex search method of Lagarias and colleagues [42]. This approach usually takes more time than Approach 1, as for each coalescence time $T_{m}$, we need to solve the corresponding equation iteratively. Furthermore, the number of equations and the computational complexity increase with the sample size, and thus Approach 2 is more suitable for small samples.

## Results

Various population growth models have been proposed to approximate the ancient population history of humans and other species. For example, Gazave et al. proposed a five-scenario model for the European population, including two stages of population bottlenecks and a very recent exponential growth [23]. The simple exponential population growth model may be the most commonly used model. It assumes a constant growth rate, which is valid when space and resources are unlimited. The exponential growth model is a good approximation for the early stage of humans, bacteria, and most populations. In cancer evolution studies, models with more parameters were developed to describe tumor growth [43]. These models are complicated by modifying growth rates with carrying capacity or other factors, *e.g.*, the logistic growth model and Gompertz model.

In this section, exponential, logistic, and Gompertz growth models are used to illustrate the usage of our proposed approach. For the exponential growth model with a growth rate γ, $N\left(t\right)=N_{0}e^{-rt}$, it is straightforward to analytically derive the expected coalescence times $ET_{m}$ [Equation (9)]. Running time using the three approaches (including the analytical approach, the finite-sum approximation, and Approach 2) was then compared for the model with the two parameters $N_{0}=100,000$ and γ=0.003. For Approach 2, two numerical methods were adopted: the bisection + interpolation method implemented in the MATLAB function fzero and the downhill simplex method implemented in the MATLAB function fminsearch. The running time was averaged over 1000 repeats run in MATLAB and is presented in Table 2. The detailed results for the logistic growth and Gompertz model are elaborated below.

**Table 2:** **Comparison of running time between different methods for three population growth models**

**Sample size**	**Method**	**Running time (second)**		
		**Exponential**	**Logistic**	**Gompertz**
10	Analytical calculation	0.000004	0.110637	–
	Finite sum approximation (Approach 1)	0.000084	0.000205	0.000204
	fzero (bisection + interpolation, Approach 2)	0.003677	0.004618	–
	fminsearch (downhill simplex, Approach 2)	0.019621	0.019983	–

50	Analytical calculation	0.000005	0.614442	–
	Finite sum approximation (Approach 1)	0.000087	0.000188	0.000208
	fzero (bisection + interpolation, Approach 2)	0.034866	0.020172	–
	fminsearch (downhill simplex, Approach 2)	0.063361	0.106250	–

100	Analytical calculation	0.000006	1.265550	–
	Finite sum approximation (Approach 1)	0.000087	0.000194	0.000206
	fzero (bisection + interpolation, Approach 2)	0.068639	0.041638	–
	fminsearch (downhill simplex, Approach 2)	0.126790	0.214588	–

500	Analytical calculation	0.000031	7.22106	–
	Finite sum approximation (Approach 1)	0.000145	0.000226	0.000209
	fzero (bisection + interpolation, Approach 2)	0.377974	0.231884	–
	fminsearch (downhill simplex, Approach 2)	0.737102	1.219030	–

*Note*: Parameter settings for the three models: exponential are listed as follows: *N*_0_ = 100,000 and *γ* = 0.003; Logsitic: *N_K_* = 10,000, *T* = 5000, and *γ* = 0.0053; Gompertz: *T* = 5000, *r* = 0.01, *α* = 0.001 and *N*_0_ = 1. For the Gompertz model, only the results of the finite-sum approximation are available.

### AFS of the logistic growth model

**Figure 1:**
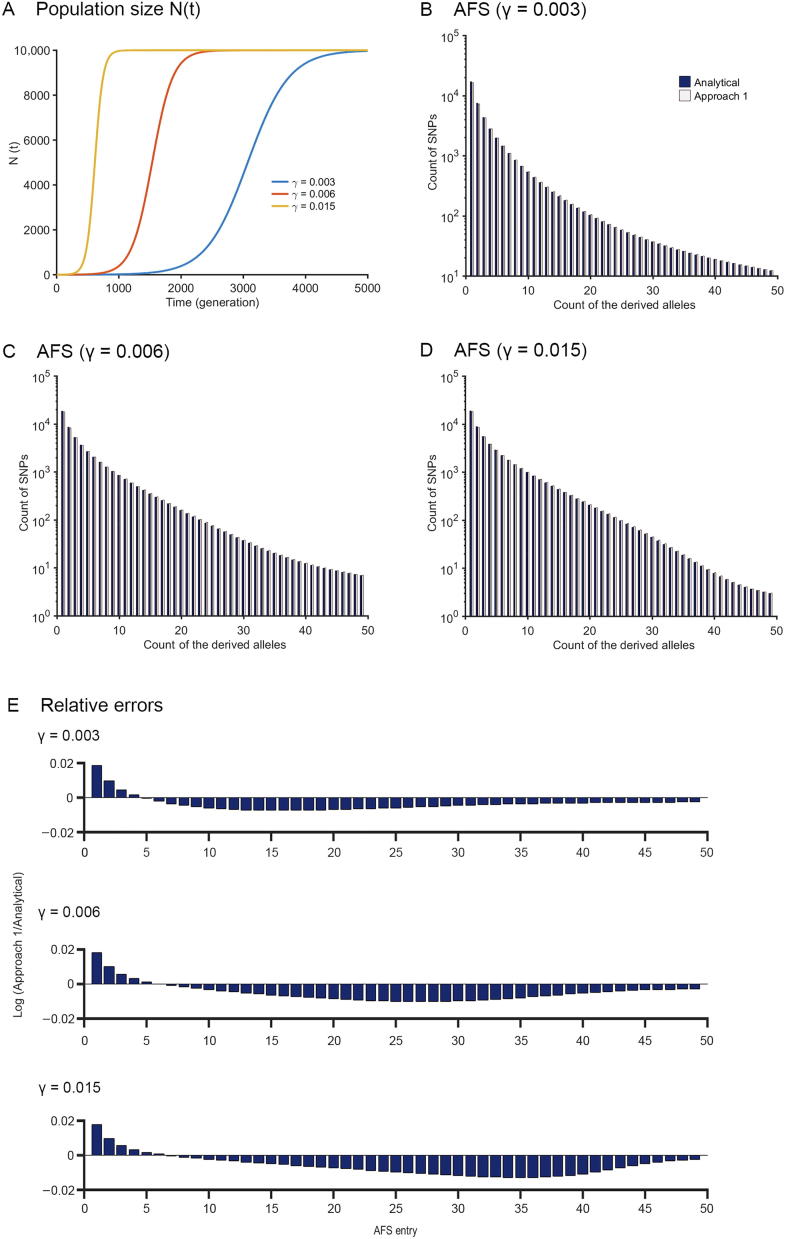
**The allele freqeuncy spectra of the logistic growth model** **A.** The population size as a function of time. **B.**–**D.** AFS of the logistic growth model for three growth rates (γ) of 0.003 (B), 0.006 (C), and 0.015 (D), respectively, with the carrying capacity $N_{k}=10,000$ and initial time T=5000. E. The relative errors of the AFS from the computational approach compared to the analytical results for the three growth rates of 0.003 (B), 0.006 (C), and 0.015 (D), respectively. AFS, allele frequency spectrum.

Compared to the exponential growth model, the logistic growth model regulates the growth rate with a factor $\left(1-\frac{N\left(\tilde{t}\right)}{N_{k}}\right)$, in which $N_{k}$ is the carrying capacity. It thus has a sigmoid shape and reaches an equilibrium size of $N_{k}$ instead of unlimited growth (Figure 1**A**). A logistic growth model is consistent with the population dynamics of many organisms and is widely used in ecological research. Let γ be the maximum population growth rate (aka, intrinsic growth rate), for a population under logistic growth, the population dynamics is described by the differential equation as below.

(13) $$\frac{dN\left(\tilde{t}\right)}{d\tilde{t}}=\gamma \left(\frac{N_{k}-N\left(\tilde{t}\right)}{N_{k}}\right)N\left(\tilde{t}\right).$$

Note that in the equation above, time is measured forward (from the past to the present), and denoted with $\tilde{t}$ to distinguish it from the backward time in other sections. The population size $N\left(\tilde{t}\right)$ follows a logistic curve,

(14) $$N\left(\tilde{t}\right)=\frac{N_{k}}{\left(1-e^{-\gamma \tilde{t}}\right)+N_{k}e^{-\gamma \tilde{t}}}.$$

After changing the variable of forward time $\tilde{t}$ to backward time t,

(15) $$N\left(t\right)=\frac{N_{k}e^{\gamma T}}{e^{\gamma T}+\left(N_{k}-1\right)e^{\gamma t}},$$

and the model includes three free parameters: $N_{k}$, γ, and T.

Given the population history function $N\left(t\right)$, the time-scaling function for the logistic growth model can be derived as follows,

(16) $$g\left(t\right)=\int_{0}^{t}\frac{1}{N\left(u\right)}du=\frac{e^{-\gamma T}\left(e^{\gamma t}-1\right)\left(N_{k}-1\right)+\gamma N_{k}t}{N_{k}\gamma },$$

and further obtained its inverse function,

(17) $$g^{-1}\left(\tau \right)=\frac{-W\left(e^{\left(N_{k}-1\right)e^{-rT}}+N_{k}\gamma \tau -\gamma T\right)+N_{k}r\tau +N_{k}-1}{r},$$

where $W\left(\cdot \right)$ is the Lambert W function, which is calculated numerically.

According to Chen and Chen [32], the expected coalescence time $ET_{m}=g^{-1}\left(\mu_{m}\right)$ can be obtained from Equation (17), which can also be calculated through Approaches 1 and 2 as described in the previous section. AFS generated from Equation (17) (“Analytical”) and Approach 1 (“Approach 1”) for$\;N_{k}=10,000$, T=5000 at three different growth rates γ=0.003,0.006,and\;0.015 were shown in Figure 1B–D. In addition, AFS was also obtained using Approach 2, and the comparison of the running time for a specific parameter setting ($N_{k}=10,000$, T=5000, and γ=0.005) for three approaches was listed in Table 2.

It can be seen that the AFS obtained by finite-sum approximation (Approach 1) is very close to that from the analytical approach (Figure 1B–D). The differences in AFS obtained using Approach 1 and that obtained using the analytical approach were further quantified by plotting $\text{log}\left(\frac{S_{i}^{approach1}}{S_{i}^{\mathrm{analytical}}}\right),\;1⩽i⩽50$ for each entry of the AFS (Figure 1E). The resulting values are within the range of $\left[-0.02,\text{\textbackslash{};}0.02\right]$, confirming the accuracy of the approximation using Approach 1.

### AFS of the Gompertz growth model

The Gompertz model is another widely used model to approximate population dynamics. It was originally proposed to explain human mortality [44] and is also used to describe the population growth of other species, including bacteria, animals, and plants [45]. The Gompertz model was found to fit well with the growth of breast cancer and 19 other tumor cell populations [46], [47], [48]. One of its forms is

(18) $$\frac{dN\left(\tilde{t}\right)}{d\tilde{t}}=\gamma \left(\tilde{t}\right)N\left(\tilde{t}\right),\;\;\text{with}\;\frac{d\gamma }{d\tilde{t}}=-\alpha \gamma \left(\tilde{t}\right).$$

And the solution of the differential equation is

(19) $$N\left(\tilde{t}\right)=N_{0}\text{exp}\left(\frac{\gamma }{\alpha }\left(1-e^{-\alpha \tilde{t}}\right)\right),$$

where γ is the initial growth rate; $N_{0}$ is the initial population size when it started to grow; and α can be viewed as the exponential decay rate of the growth rate.

**Figure 2:**
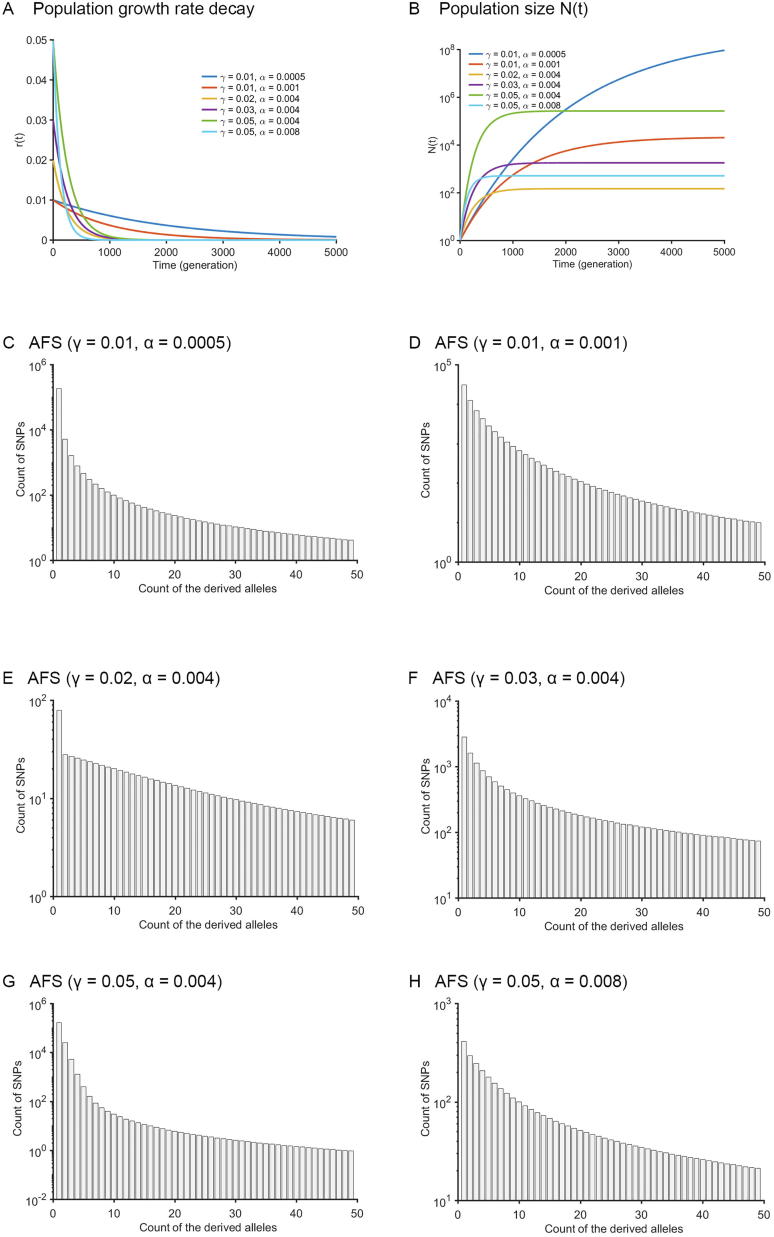
**The allele frequency spectra of the Gompertz model** **A.** The population growth rate as a function of time. **B.** The population size as a function of time. **C.–H.** AFS of the Gompertz model for six settings with different combination of growth rate (γ) and its exponential decay rate (α): *γ* = 0.01, *α* = 0.0005 (C); *γ* = 0.01, *α* = 0.001 (D); *γ* = 0.02, *α* = 0.004 (E); *γ* = 0.03, *α* = 0.004 (F); *γ* = 0.05, *α* = 0.004 (G), and *γ* = 0.05, *α* = 0.008 (H), with initial population size $N_{0}=1$ and time T=5000.

It is unfeasible to derive the time-scaling function $g\left(t\right)$ and its inverse function $g^{-1}\left(t\right)$ for the Gompertz model. Therefore, there is no analytical calculation or numerical solution (Approach 2) of the coalescence times for the Gompertz model. In Figure 2A and B, the growth rates and population size trajectories as a function of time were shown for six parameter settings: γ=0.01,α=0.0005;γ=0.01,α=0.001;γ=0.02,α=0.004;γ=0.03,α=0.004;γ=0.05,α=0.004; and γ=0.05,α=0.008. The corresponding AFS for n=50 haplotypes at these six parameter settings are presented in Figure 2C–H. The running time of Approach 1 for a specific parameter setting (T=5000, r=0.01, α=0.001 and $N_{0}=1$) and with different sample sizes (10, 50, 100, and 500) is presented in Table 2.

### Comparison of computing time of different approaches

We compared the computing times to construct the coalescence times $T_{m},\;1⩽m<n,$ using Approach 1 (finite-sum approximation), Approach 2, and the analytical approach. For Approach 2, we used two methods for solving the non-linear equations, including the combination of bisection and interpolation (bisection + interpolation; implemented in the MATLAB function fzero) and the downhill simplex (implemented in fminsearch) methods. All the comparisons are run in MATLAB for three population growth models: the exponential growth, logistic growth, and Gompertz growth model. The running time for constructing the coalescence times was recorded for four sample sizes (n=10,50,100, and 500) and averaged over 1000 repeats, as listed in Table 2 (in seconds).

A trend in Table 2 worth noting is that the finite-sum approximation runs very fast. The running time of finite-sum approximation is close to that of the analytical calculation, nearly of the same magnitude, and much shorter than that of numerical approaches (Approach 2). The only outlier is the logistic model, for which the finite-sum approximation runs much faster than the analytical approach. This is because the analytical form of the $g\left(t\right)$ function for the logistic model consists of the Lambert W function, which is calculated numerically and is time-consuming.

Second, the running time of the finite-sum approximation approach is nearly constant with increasing sample size n. As we mentioned above, the computational complexity is $O\left(1\right)$, and thus, it is insensitive to the sample size. This guarantees the computational efficiency of the approach when the sample size becomes large, enabling its application to large-sample data analysis.

The numerical approach for solving the $g\left(t\right)$ function (Approach 2) also works efficiently but is more time-consuming than the finite-sum approximation approach for all three population growth models. Furthermore, the running time increases with the sample size n, as the number of nonlinear equations to solve increases linearly with n.

## Conclusion

AFS is informative for population genetic inference. Various AFS-based methods have been developed for inferring population histories and detecting natural selection in the past years. They have gained popularity with the abundance of genomic sequencing data (*e.g.*, [3], [5], [49], [7], [8], [9], [10]). Compared with the diffusion-based AFS methods that require approximation of the solutions with numerical approaches, modeling AFS using coalescent theory is computationally efficient. Most population genetic inference methods using the coalescent likelihood require computationally intensive algorithms for parameter estimation, such as importance sampling or Markov chain Monte Carlo, while the coalescent-based AFS methods only depend on the expected coalescence times, which guarantee the analytical form [13], [2], [3].

The coalescent-based AFS methods have shortcomings. First, for large samples it is impossible to obtain accurate calculations due to numerical overflow of large coefficients in the hypergeometric series. Second, it is difficult to derive the coalescent-based AFS for complex population histories, which limits its application to simple growth models, such as the exponential growth and n-epoch models. Chen and Chen [32] showed that for complex demography, we can obtain the expected coalescence times through a linear Taylor expansion approximation, which involves the time-scaling function $g\left(t\right)$ and its inverse function $g^{-1}\left(t\right)$. The analytical equations of coalescence times derived using this approach are in a simple form and can successfully overcome the numerical issue for large samples. Furthermore, the time-scaling scheme is technically applicable to arbitrary complex population histories. However, in practice, the analytical forms of the time-scaling function $g\left(t\right)$ and its inverse function are not achievable for many cases, limiting the applications. For example, in the study of cancer cell growth, various population growth models in complex form were proposed to describe the dynamics of cancer cells [43], for which the analytical form of AFS is difficult to derive. In this paper, we propose a computational approach, the finite-sum approximation, which efficiently solves the problem of Chen and Chen [32] when the analytical form of the time-scaling function $g\left(t\right)$ and its inverse function $g^{-1}\left(t\right)$ are not derivable.

We apply the computational approach to three widely used models, including the exponential, logistic, and Gompertz growth models to demonstrate its performance. As shown in the Results section, the finite-sum approximation approach is computationally very efficient, and the running time is nearly on the magnitude of that of the analytical approach. Furthermore, the computational time does not increase linearly with the sample size, ensuring its efficiency for AFS of large sample sizes. This is especially attractive for the flexibility to tackle a complex population history that is intractable by using the analytical approach, for example, the Gompertz growth model shown in Table 2. The computational approach presented in this paper is applicable to single populations with arbitrary complex varying size and significantly enables the application of the coalescent-based AFS approaches to population genetic inference in the genomic sequencing era. However, we should note that using the proposed computational approach to model the joint AFS of multiple populations with arbitrary population size changes and gene flows remains challenging and will be addressed in future work.

## Availability

Software for implementing the algorithm can be downloaded from the lab webpage at http://chenlab.big.ac.cn/software/.

## Author’s contributions

HC designed the study, developed the method, and performed the analysis. HC wrote the manuscript and approved the final manuscript.

## Competing interests

The author has declared no competing interests.

## References

[1] Kimura M. (1955). Solution of a process of random genetic drift with a continuous model. Proc Natl Acad Sci U S A.

[2] Fu Y.X. (1995). Statistical properties of segregating sites. Theor Popul Biol.

[3] Griffiths R.C., Tavaré S. (1998). The age of a mutation in a general coalescent tree. Stoch Model.

[4] Sawyer S.A., Hartl D.L. (1992). Population genetics of polymorphism and divergence. Genetics.

[5] Bustamante C.D., Wakeley J., Sawyer S., Hartl D.L. (2001). Directional selection and the site-frequency spectrum. Genetics.

[6] Wooding S.P., Watkins W.S., Bamshad M.J., Dunn D.M., Weiss R.B., Jorde L.B. (2002). DNA sequence variation in a 3.7-kb noncoding sequence 5′ of the CYP1A2 gene: implications for human population history and natural selection. Am J Hum Genet.

[7] Polanski A., Kimmel M. (2003). New explicit expressions for relative frequencies of single-nucleotide polymorphisms with application to statistical inference on population growth. Genetics.

[8] Marth G.T., Czabarka E., Murvai J., Sherry S.T. (2004). The allele frequency spectrum in genome-wide human variation data reveals signals of differential demographic history in three large world populations. Genetics.

[9] Williamson S.H., Hernandez R., Fledel-Alon A., Zhu L., Nielsen R., Bustamante C.D. (2005). Simultaneous inference of selection and population growth from patterns of variation in the human genome. Proc Natl Acad Sci U S A.

[10] Gutenkunst R.N., Hernandez R.D., Williamson S.H., Bustamante C.D. (2009). Inferring the joint demographic history of multiple populations from multidimensional SNP frequency data. PLoS Genet.

[11] Lukic S., Hey J., Chen K. (2011). Non-equilibrium allele frequency spectra via spectral methods. Theor Popul Biol.

[12] Zivkovic D., Stephan W. (2011). Analytical results on the neutral non-equilibrium allele frequency spectrum based on diffusion theory. Theor Popul Biol.

[13] Chen H. (2012). The joint allele frequency spectrum of multiple populations: a coalescent theory approach. Theor Popul Biol.

[14] Excoffier L., Dupanloup I., Huerta-Sanchez E., Sousa V.C., Foll M. (2013). Robust demographic inference from genomic and SNP data. PLoS Genet.

[15] Gao F., Keinan A. (2016). Inference of super-exponential human population growth via efficient computation of the site frequency spectrum for generalized models. Genetics.

[16] Bhaskar A., Wang Y.X., Song Y.S. (2015). Efficient inference of population size histories and locus-specific mutation rates from large-sample genomic variation data. Genome Res.

[17] Liu X.M., Fu Y.X. (2015). Exploring population size changes using SNP frequency spectra. Nat Genet.

[18] Wooding S., Rogers A. (2002). The matrix coalescent and an application to human single-nucleotide polymorphisms. Genetics.

[19] Evans S.N., Shvets Y., Slatkin M. (2007). Non-equilibrium theory of the allele frequency spectrum. Theor Popul Biol.

[20] Marth G., Schuler G., Yeh R., Davenport R., Agarwala R., Church D. (2003). Sequence variations in the public human genome data reflect a bottlenecked population history. Proc Natl Acad Sci U S A.

[21] Keinan A., Mullikin J.C., Patterson N., Reich D. (2007). Measurement of the human allele frequency spectrum demonstrates greater genetic drift in East Asians than in Europeans. Nat Genet.

[22] Gravel S., Henn B.M., Gutenkunst R.N., Indap A.R., Marth G.T., Clark A.G. (2011). Demographic history and rare allele sharing among human populations. Proc Natl Acad Sci U S A.

[23] Gazave E., Ma L., Chang D., Coventry A., Gao F., Muzny D. (2014). Neutral genomic regions refine models of recent rapid human population growth. Proc Natl Acad Sci U S A.

[24] Chen H. (2015). Population genetic studies in the genomic sequencing era. Zool Res.

[25] Chen H. (2013). Intercoalescence time distribution of incomplete gene genealogies in temporally varying populations, and applications in population genetic inference. Ann Hum Genet.

[26] Polanski A., Bobrowski A., Kimmel M. (2003). A note on distributions of times to coalescence, under time-dependent population size. Theor Popul Biol.

[27] Coventry A., Bull-Otterson L.M., Liu X., Clark A.G., Maxwell T.J., Crosby J. (2010). Deep resequencing reveals excess rare recent variants consistent with explosive population growth. Nat Commun.

[28] Chen H., Hey J., Chen K. (2015). Inferring very recent population growth rate from population-scale sequencing data: using a large-sample coalescent estimator. Mol Biol Evol.

[29] Hudson R.R. (2002). Generating samples under a Wright-Fisher neutral model of genetic variation. Bioinformatics.

[30] Nelson M.R., Wegmann D., Ehm M.G., Kessner D., St Jean P., Verzilli C. (2012). An abundance of rare functional variants in 202 drug target genes sequenced in 14,002 people. Science.

[31] Tennessen J.A., Bigham A.W., O'Connor T.D., Fu W., Kenny E.E., Gravel S. (2012). Evolution and functional impact of rare coding variation from deep sequencing of human exomes. Science.

[32] Chen H., Chen K. (2013). Asymptotic distributions of coalescence times and ancestral lineage numbers for populations with temporally varying size. Genetics.

[33] Griffiths R.C. (1984). Asymptotic line-of-descent distributions. J Math Biol.

[34] Griffiths R.C., Tavare S. (1994). Sampling theory for neutral alleles in a varying environment. Philos Trans R Soc Lond B Biol Sci.

[35] Donnelly P., Tavare S. (1995). Coalescents and genealogical structure under neutrality. Annu Rev Genet.

[36] Nordborg M., Balding D., Bishop M., Cannings C. (2001). Coalescent theory. Handbook of statistical genetics.

[37] Feller W. (2008). An introduction to probability theory and its applications.

[38] Griffiths R.C. (2006). Coalescent lineage distributions. Adv Appl Probab.

[39] Kingman J.F.C. (1982). The coalescent. Stoch Process Their Appl.

[40] Press W.H., Teukolsky S.A., Vetterling W.T., Flannery B.P. (2007). Numerical recipes: the art of scientific computing.

[41] Brent R.P. (2013). Algorithms for minimization without derivatives.

[42] Lagarias J.C., Reeds J.A., Wright M.H., Wright P.E. (1998). Convergence properties of the Nelder-Mead simplex method in low dimensions. SIAM J Optim.

[43] Benzekry S., Lamont C., Beheshti A., Tracz A., Ebos J.M., Hlatky L. (2014). Classical mathematical models for description and prediction of experimental tumor growth. PLoS Comput Biol.

[44] Gompertz B. (1825). On the nature of the function expressive of the law of human mortality, and on a new mode of determining the value of life contingencies. Philos Trans R Soc Lond.

[45] Tjorve K.M.C., Tjorve E. (2017). The use of Gompertz models in growth analyses, and new Gompertz-model approach: an addition to the Unified-Richards family. PLoS One.

[46] Laird A.K. (1964). Dynamics of tumour growth. Br J Cancer.

[47] Norton L., Simon R., Brereton H.D., Bogden A.E. (1976). Predicting the course of Gompertzian growth. Nature.

[48] Norton L. (1988). A Gompertzian model of human breast cancer growth. Cancer Res.

[49] Nielsen R., Williamson S., Kim Y., Hubisz M.J., Clark A.G., Bustamante C. (2005). Genomic scans for selective sweeps using SNP data. Genome Res.

